# Supplementary material for: Intermolecular Halogen Bond Detected in Racemic and Optically Pure N-C Axially Chiral 3-(2-Halophenyl)quinazoline-4-thione Derivatives
Source: Molecules. 2022 Apr 6;27(7):2369. doi: 10.3390/molecules27072369 (PMC9000658; doi:10.3390/molecules27072369)
Supplement: Supplementary file 1 [file molecules-27-02369-s001.zip › molecules-1643757-supplementary.pdf]

Supplementary Materials

Intermolecular halogen bond detected in racemic and optically pure N-C axially chiral 3-(2-halophenyl)quinazoline-4-thione derivatives

Ryosuke Matsui,[1] Erina Niijima,[1] Tomomi Imai,[1] Hiroyuki Kobayashi,[2] Akiko Hori,[2] Azusa Sato,[3] Yuko Nakamura[3] and Osamu Kitagawa*[1]

[1] Department of Applied Chemistry (Japanese Association of Bio-intelligence for Well-being), Shibaura institute of Technology, 3-7-5 Toyosu, Kohto-ku, Tokyo, 135-8548, Japan.

[2] Graduate School of Engineering and Science, Shibaura Institute of Technology, 307 Fukasaku, Minuma-ku, Saitama 337-8570, Japan.

[3] Division of Basic Sciences, Center for Medical and Nursing Education, Tokyo Women's Medical University, 8-1 Kawada-cho, Shinjuku-ku, 162-8666 Tokyo, Japan

AUTHOR E-MAIL ADDRESS: kitagawa@shibaura-it.ac.jp

(Contents)

Copies of ^{1}H- and ^{13}C-NMR chart of compound **2c** S2

CheckCIF of ***rac*-2a, *rac*-2b, (*P*)-2b** and ***rac*-2c** S3-S18

Hirshfeld Surface analyses of ***rac*-2a**, **(*P*)-2a**, ***rac*-2b**, **(*P*)-2b** and ***rac*-2c** S-19-S20

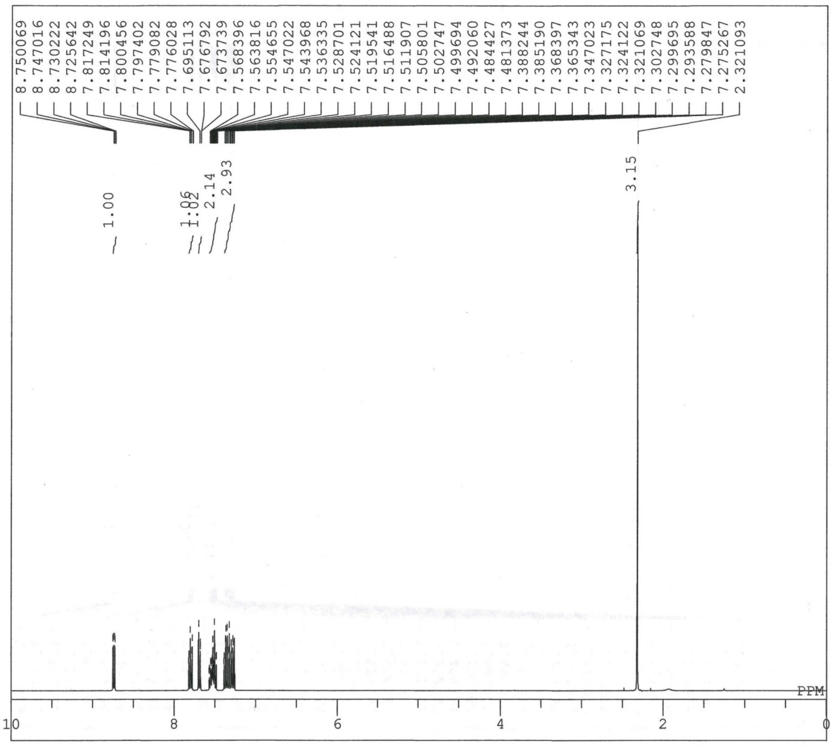

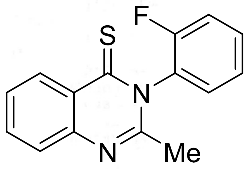

2c

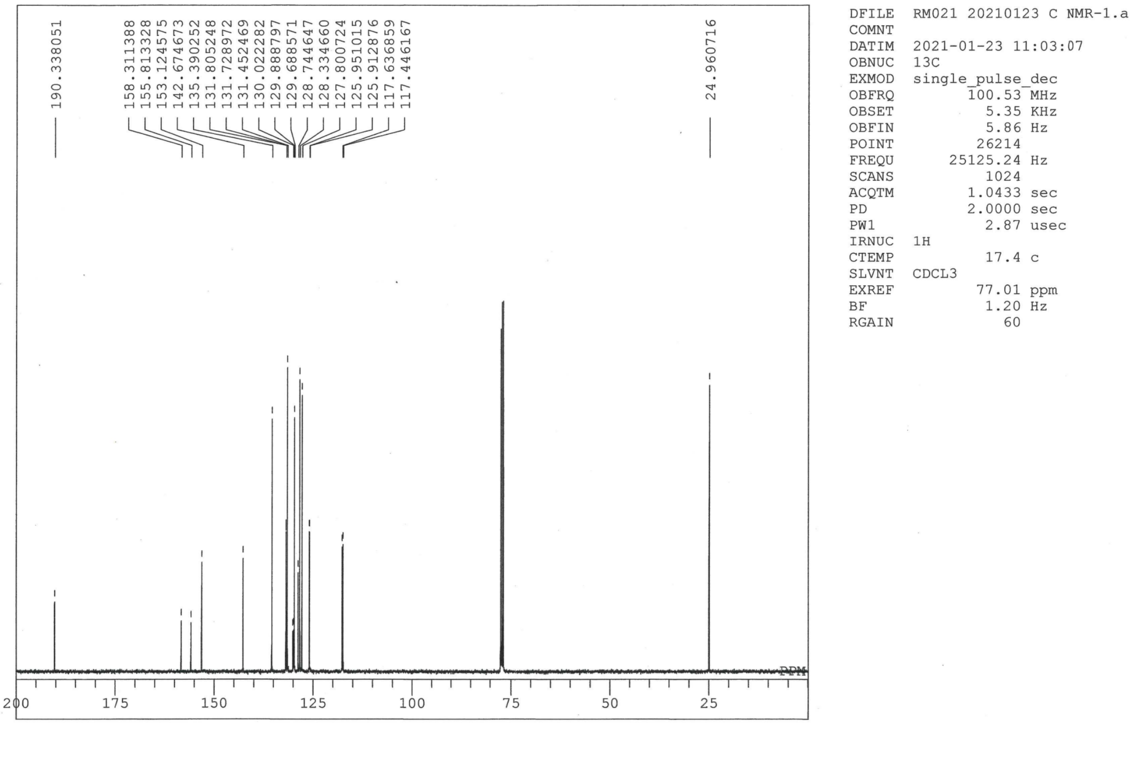

Figure S1. ^{1}H-NMR and ^{13}C-NMR spectra of compound **2c**.

CheckCIF/PLATON report

***rac*-2a**

Structure factors have been supplied for datablock(s) en191206b_1_Pbca_a

THIS REPORT IS FOR GUIDANCE ONLY. IF USED AS PART OF A REVIEW PROCEDURE FOR PUBLICATION, IT SHOULD NOT REPLACE THE EXPERTISE OF AN EXPERIENCED CRYSTALLOGRAPHIC REFEREE.

No syntax errors found. CIF dictionary Interpreting this report

Table S1. Datablock: en191206b_1_Pbca_a

Bond precision:	C-C = 0.0030 A	Wavelength=0.71073	
Cell:	a=13.9862(4)	b=11.8103(4)	c=16.7044(5)
	alpha=90	beta=90	gamma=90
Temperature:	100 K		

	Calculated	Reported
Volume	2759.25(15)	2759.25(15)
Space group	P b c a	P b c a
Hall group	-P 2ac 2ab	-P 2ac 2ab
Moiety formula	C15 H11 Br N2 S	C15 H11 Br N2 S
Sum formula	C15 H11 Br N2 S	C15 H11 Br N2 S
Mr	331.22	331.23
Dx,g cm-3	1.595	1.595
Z	8	8
Mu (mm-1)	3.117	3.117
F000	1328.0	1328.0
F000’	1327.09	
h,k,lmax	16,14,19	16,14,19
Nref	2437	2435
Tmin,Tmax	0.502,0.746	0.500,0.760
Tmin’	0.447	

Correction method= # Reported T Limits: Tmin=0.500 Tmax=0.760
AbsCorr = MULTI-SCAN

Data completeness= 0.999 Theta(max)= 25.026

R(reflections)= 0.0203(2147) wR2(reflections)= 0.0496(2435)

S = 1.030 Npar= 173

Table S2. The following ALERTS were generated. Each ALERT has the format
test-name_ALERT_alert-type_alert-level.
Click on the hyperlinks for more details of the test.

Alert level C

PLAT431_ALERT_2_C Short Inter HL..A Contact Br1	..S1	.	3.41 Ang.
	1/2-x,1/2+y,z =		8_665 Check

Alert level G

PLAT380_ALERT_4_G Incorrectly? Oriented X(sp2)-Methyl Moiety	C9	Check
PLAT883_ALERT_1_G No Info/Value for _atom_sites_solution_primary .	Please Do	!
PLAT909_ALERT_3_G Percentage of I>2sig(I) Data at Theta(Max) Still	76%	Note
PLAT910_ALERT_3_G Missing # of FCF Reflection(s) Below Theta(Min).	1	Note
PLAT933_ALERT_2_G Number of HKL-OMIT Records in Embedded .res File	1	Note
PLAT978_ALERT_2_G Number C-C Bonds with Positive Residual Density.	13	Info

0 **ALERT level A** = Most likely a serious problem - resolve or explain
0 **ALERT level B** = A potentially serious problem, consider carefully
1 **ALERT level C** = Check. Ensure it is not caused by an omission or oversight
6 **ALERT level G** = General information/check it is not something unexpected

1 ALERT type 1 CIF construction/syntax error, inconsistent or missing data
3 ALERT type 2 Indicator that the structure model may be wrong or deficient
2 ALERT type 3 Indicator that the structure quality may be low
1 ALERT type 4 Improvement, methodology, query or suggestion
0 ALERT type 5 Informative message, check

It is advisable to attempt to resolve as many as possible of the alerts in all categories. Often the minor alerts point to easily fixed oversights, errors and omissions in your CIF or refinement strategy, so attention to these fine details can be worthwhile. In order to resolve some of the more serious problemsit may be necessary to carry out additional measurements or structure refinements. However, the purpose of your study may justify the reported deviations and the more serious of these should normally be commented upon in the discussion or experimental section of a paper or in the "special_details" fields of the CIF. checkCIF was carefully designed to identify outliers and unusual parameters, but every test has its limitations and alerts that are not important in a particular case may appear. Conversely, the absence of alerts does not guarantee there are no aspects of the results needingattention. It is up to the individual to critically assess their own results and, if necessary, seek expert advice.

Publication of your CIF in IUCr journals

A basic structural check has been run on your CIF. These basic checks will be run on all CIFs submitted for publication in IUCr journals (*Acta Crystallographica*, *Journal of Applied Crystallography*, *Journal of Synchrotron Radiation*); however, if you intend to submit to *Acta Crystallographica Section C* or *E* or *IUCrData*, you should make sure that full publication checks arerun on the final version of your CIF prior to submission.

Publication of your CIF in other journals

Please refer to the *Notes for Authors* of the relevant journal for any special instructions relating to CIFsubmission.

PLATON version of 18/12/2021; check.def file version of 18/12/2021

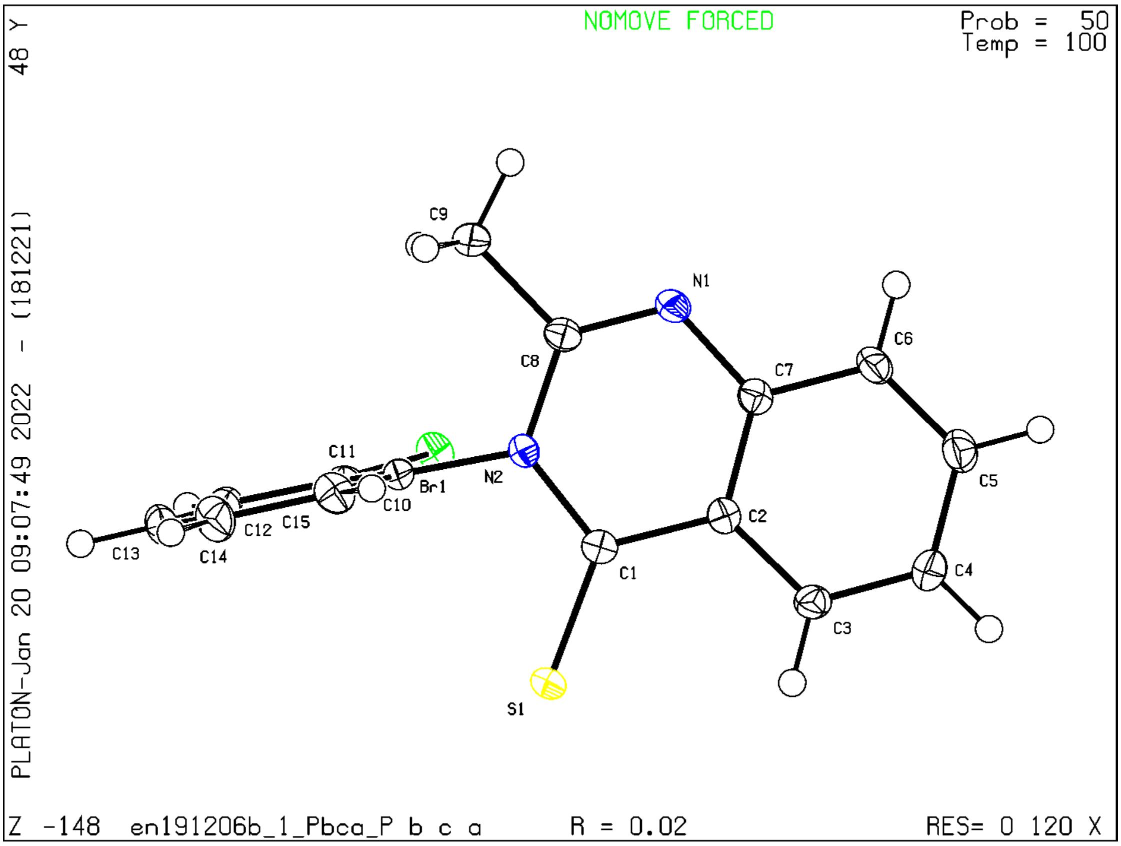

Figure S2. **Datablock en191206b_1_Pbca_a** - ellipsoid plot (**rac-2a**).

***rac*-2b**

Structure factors have been supplied for datablock(s) Pbca2_a

THIS REPORT IS FOR GUIDANCE ONLY. IF USED AS PART OF A REVIEW PROCEDURE FORPUBLICATION, IT SHOULD NOT REPLACE THE EXPERTISE OF AN EXPERIENCED CRYSTALLOGRAPHIC REFEREE.

No syntax errors found. CIF dictionary Interpreting this report

Table S3. Datablock: Pbca2_a

Bond precision:	C-C = 0.0020 A		Wavelength=0.71073
Cell:	a=14.1210(16)	b=11.5800(14)	c=16.6932(17)
	alpha=90	beta=90	gamma=90
Temperature:	100 K		

	Calculated	Reported
Volume	2729.7(5)	2729.7(5)
Space group	P b c a	P b c a
Hall group	-P 2ac 2ab	-P 2ac 2ab
Moiety formula	C15 H11 Cl N2 S	C15 H11 Cl N2 S
Sum formula	C15 H11 Cl N2 S	C15 H11 Cl N2 S
Mr	286.77	286.77
Dx,g cm-3	1.396	1.396
Z	8	8
Mu (mm-1)	0.419	0.419
F000	1184.0	1184.0
F000’	1186.50	
h,k,lmax	16,13,19	16,13,19
Nref	2406	2403
Tmin,Tmax	0.875,0.899	0.700,0.900
Tmin’	0.875	

Correction method= # Reported T Limits: Tmin=0.700 Tmax=0.900
AbsCorr = MULTI-SCAN

Data completeness= 0.999 Theta(max)= 25.023

R(reflections)= 0.0297(2261) wR2(reflections)= 0.0840(2403)

S = 1.054 Npar= 173

Table S4. The following ALERTS were generated. Each ALERT has the format
test-name_ALERT_alert-type_alert-level.
Click on the hyperlinks for more details of the test.

Alert level C

PLAT230_ALERT_2_C Hirshfeld Test Diff for	S1	--C1	.	6.1	s.u.
PLAT934_ALERT_3_C Number of (Iobs-Icalc)/Sigma(W) > 10 Outliers ..			1 Check		

Alert level G

PLAT883_ALERT_1_G No Info/Value for _atom_sites_solution_primary .	Please	Do !
PLAT909_ALERT_3_G Percentage of I>2sig(I) Data at Theta(Max) Still	88%	Note
PLAT910_ALERT_3_G Missing # of FCF Reflection(s) Below Theta(Min).	3	Note
PLAT933_ALERT_2_G Number of HKL-OMIT Records in Embedded .res File	3	Note
PLAT965_ALERT_2_G The SHELXL WEIGHT Optimisation has not Converged	Please	Check
PLAT978_ALERT_2_G Number C-C Bonds with Positive Residual Density.	8 Info	

0 **ALERT level A** = Most likely a serious problem - resolve or explain
0 **ALERT level B** = A potentially serious problem, consider carefully
2 **ALERT level C** = Check. Ensure it is not caused by an omission or oversight
6 **ALERT level G** = General information/check it is not something unexpected

1 ALERT type 1 CIF construction/syntax error, inconsistent or missing data
4 ALERT type 2 Indicator that the structure model may be wrong or deficient
3 ALERT type 3 Indicator that the structure quality may be low
0 ALERT type 4 Improvement, methodology, query or suggestion
0 ALERT type 5 Informative message, check

It is advisable to attempt to resolve as many as possible of the alerts in all categories. Often the minor alerts point to easily fixed oversights, errors and omissions in your CIF or refinement strategy, so attention to these fine details can be worthwhile. In order to resolve some of the more serious problemsit may be necessary to carry out additional measurements or structure refinements. However, the purpose of your study may justify the reported deviations and the more serious of these should normally be commented upon in the discussion or experimental section of a paper or in the "special_details" fields of the CIF. checkCIF was carefully designed to identify outliers and unusual parameters, but every test has its limitations and alerts that are not important in a particular case may appear. Conversely, the absence of alerts does not guarantee there are no aspects of the results needingattention. It is up to the individual to critically assess their own results and, if necessary, seek expert advice.

Publication of your CIF in IUCr journals

A basic structural check has been run on your CIF. These basic checks will be run on all CIFs submitted for publication in IUCr journals (*Acta Crystallographica*, *Journal of Applied Crystallography*, *Journal of Synchrotron Radiation*); however, if you intend to submit to *Acta Crystallographica Section C* or *E* or *IUCrData*, you should make sure that full publication checks arerun on the final version of your CIF prior to submission.

Publication of your CIF in other journals

Please refer to the *Notes for Authors* of the relevant journal for any special instructions relating to CIFsubmission.

PLATON version of 18/12/2021; check.def file version of 18/12/2021

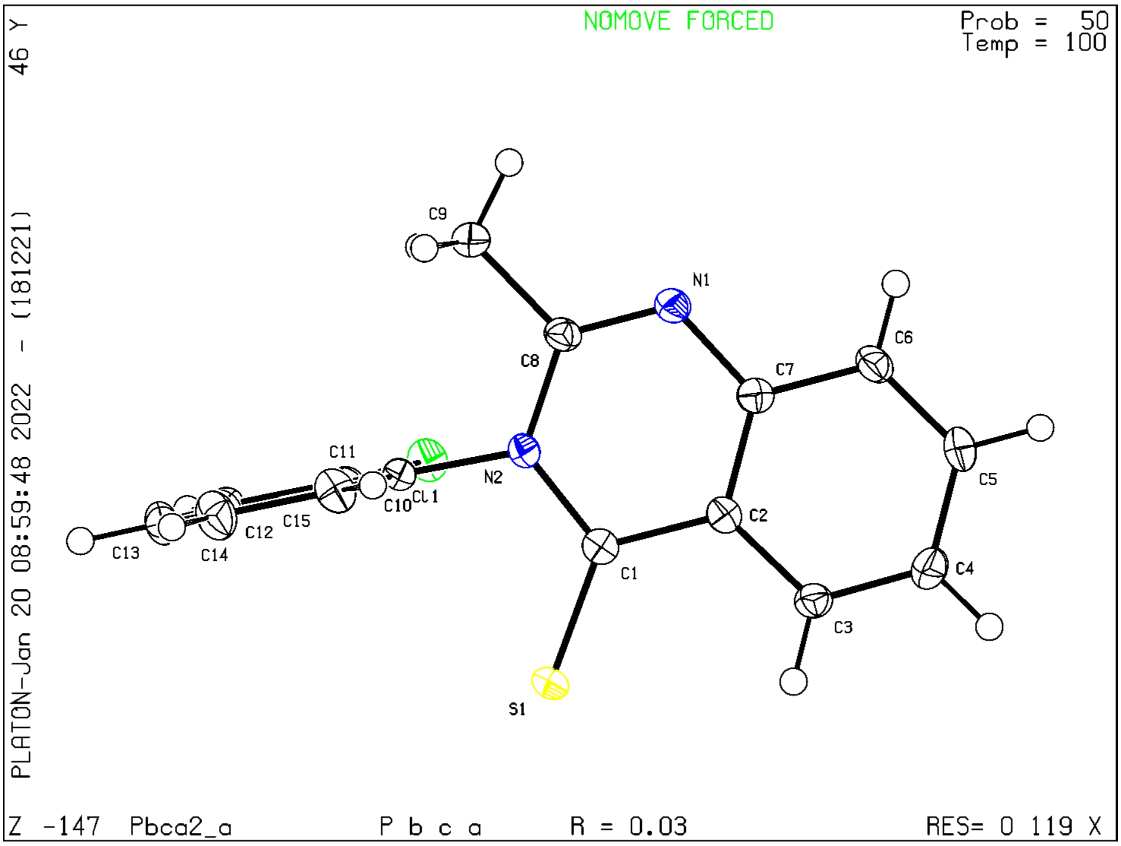

Figure S3. Datablock Pbca2_a - ellipsoid plot (**rac-2b**).

(*P*)-2b

Structure factors have been supplied for datablock(s) P21_a

THIS REPORT IS FOR GUIDANCE ONLY. IF USED AS PART OF A REVIEW PROCEDURE FORPUBLICATION, IT SHOULD NOT REPLACE THE EXPERTISE OF AN EXPERIENCED CRYSTALLOGRAPHIC REFEREE.

No syntax errors found. CIF dictionary Interpreting this report

Table S5. Datablock: P21_a

Bond precision: C-C = 0.0041 A Wavelength=0.71073

Cell:	a=8.3918(7)	b=16.9928(15)	c=10.0448(10)
	alpha=90	beta=111.646	gamma=90
Temperature:	100 K		

	Calculated	Reported
Volume	1331.4(2)	1331.4(2)
Space group	P 21	P 21
Hall group	P 2yb	P 2yb
Moiety formula	C15 H11 Cl N2 S	C15 H11 Cl N2 S
Sum formula	C15 H11 Cl N2 S	C15 H11 Cl N2 S
Mr	286.77	286.77
Dx,g cm-3	1.431	1.431
Z	4	4
Mu (mm-1)	0.429	0.429
F000	592.0	592.0
F000’	593.25	
h,k,lmax	9,20,11	9,20,11
Nref	4690 [2431]	4472
Tmin,Tmax	0.897,0.934	0.770,0.940
Tmin’	0.873	

Correction method= # Reported T Limits: Tmin=0.770 Tmax=0.940
AbsCorr = MULTI-SCAN

Data completeness= 1.84/0.95 Theta(max)= 25.026

R(reflections)= 0.0256(4381) wR2(reflections)= 0.0631(4472)

S = 1.060 Npar= 345

Table S6. The following ALERTS were generated. Each ALERT has the format **test-name_ALERT_alert-type_alert-level**.
Click on the hyperlinks for more details of the test.

Alert level C

PLAT089_ALERT_3_C Poor Data / Parameter Ratio (Zmax < 18)		7.03	Note
PLAT340_ALERT_3_C Low Bond Precision on C-C Bonds	0.00407		Ang.
PLAT911_ALERT_3_C Missing FCF Refl Between Thmin & STh/L=	0.595	4	Report

Alert level G

PLAT380_ALERT_4_G Incorrectly? Oriented X(sp2)-Methyl Moiety	C24	Check
PLAT883_ALERT_1_G No Info/Value for _atom_sites_solution_primary .	Please	Do !
PLAT909_ALERT_3_G Percentage of I>2sig(I) Data at Theta(Max) Still	96%	Note
PLAT933_ALERT_2_G Number of HKL-OMIT Records in Embedded .res File	2	Note
PLAT978_ALERT_2_G Number C-C Bonds with Positive Residual Density.	2	Info

0 **ALERT level A** = Most likely a serious problem - resolve or explain
0 **ALERT level B** = A potentially serious problem, consider carefully
3 **ALERT level C** = Check. Ensure it is not caused by an omission or oversight
5 **ALERT level G** = General information/check it is not something unexpected

1 ALERT type 1 CIF construction/syntax error, inconsistent or missing data
2 ALERT type 2 Indicator that the structure model may be wrong or deficient
4 ALERT type 3 Indicator that the structure quality may be low
1 ALERT type 4 Improvement, methodology, query or suggestion
0 ALERT type 5 Informative message, check

It is advisable to attempt to resolve as many as possible of the alerts in all categories. Often the minor alerts point to easily fixed oversights, errors and omissions in your CIF or refinement strategy, so attention to these fine details can be worthwhile. In order to resolve some of the more serious problemsit may be necessary to carry out additional measurements or structure refinements. However, the purpose of your study may justify the reported deviations and the more serious of these should normally be commented upon in the discussion or experimental section of a paper or in the "special_details" fields of the CIF. checkCIF was carefully designed to identify outliers and unusual parameters, but every test has its limitations and alerts that are not important in a particular case may appear. Conversely, the absence of alerts does not guarantee there are no aspects of the results needingattention. It is up to the individual to critically assess their own results and, if necessary, seek expert advice.

Publication of your CIF in IUCr journals

A basic structural check has been run on your CIF. These basic checks will be run on all CIFs submitted for publication in IUCr journals (*Acta Crystallographica*, *Journal of Applied Crystallography*, *Journal of Synchrotron Radiation*); however, if you intend to submit to *Acta Crystallographica Section C* or *E* or *IUCrData*, you should make sure that full publication checks arerun on the final version of your CIF prior to submission.

Publication of your CIF in other journals

Please refer to the *Notes for Authors* of the relevant journal for any special instructions relating to CIFsubmission.

PLATON version of 18/12/2021; check.def file version of 18/12/2021

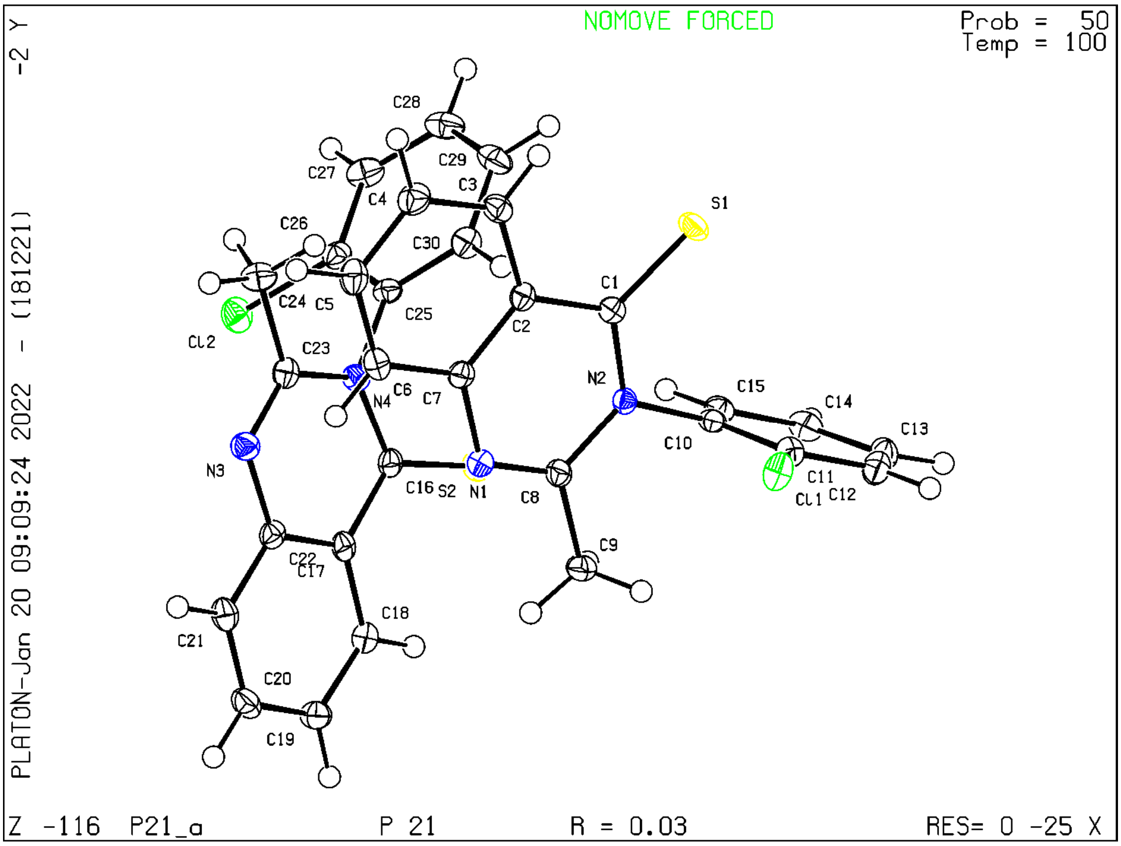

Figure S4. **Datablock P21_a** - ellipsoid plot [(*P*)-**2b**].

***rac*-2c**

Structure factors have been supplied for datablock(s) hk201126b

THIS REPORT IS FOR GUIDANCE ONLY. IF USED AS PART OF A REVIEW PROCEDURE FORPUBLICATION, IT SHOULD NOT REPLACE THE EXPERTISE OF AN EXPERIENCED CRYSTALLOGRAPHIC REFEREE.

No syntax errors found. CIF dictionary Interpreting this report

Table S7. Datablock: hk201126b

Bond precision:	C-C = 0.0030 A	Wavelength=0.71073	
Cell:	a=13.3633(19)<br>alpha=90	b=14.1656(18)<br>beta=114.812(4)	c=14.7771(18)<br>gamma=90
Temperature:	100 K		

	Calculated	Reported
Volume	2539.1(6)	2539.1(6)
Space group	C 2/c	C 2/c
Hall group	-C 2yc	-C 2yc
Moiety formula	C15 H11 F N2 S	C15 H11 F N2 S
Sum formula	C15 H11 F N2 S	C15 H11 F N2 S
Mr	270.32	270.32
Dx,g cm-3	1.414	1.414
Z	8	8
Mu (mm-1)	0.253	0.253
F000	1120.0	1120.0
F000’	1121.43	
h,k,lmax	15,16,17	15,16,17
Nref	2247	2245
Tmin,Tmax	0.937,0.955	0.830,0.960
Tmin’	0.930	

Correction method= # Reported T Limits: Tmin=0.830 Tmax=0.960
AbsCorr = MULTI-SCAN

Data completeness= 0.999 Theta(max)= 25.024

R(reflections)= 0.0352(2010) wR2(reflections)= 0.1121(2245)

S = 1.124 Npar= 173

Table S8. The following ALERTS were generated. Each ALERT has the format **test-name_ALERT_alert-type_alert-level**.
Click on the hyperlinks for more details of the test.

Alert level C

PLAT911_ALERT_3_C Missing FCF Refl Between Thmin & STh/L= 0.595 3 Report
PLAT934_ALERT_3_C Number of (Iobs-Icalc)/Sigma(W) > 10 Outliers .. 1 Check

Alert level G

PLAT883_ALERT_1_G No Info/Value for _atom_sites_solution_primary . Please Do !
PLAT909_ALERT_3_G Percentage of I>2sig(I) Data at Theta(Max) Still 76% Note
PLAT965_ALERT_2_G The SHELXL WEIGHT Optimisation has not Converged Please Check
PLAT978_ALERT_2_G Number C-C Bonds with Positive Residual Density. 3 Info

0 **ALERT level A** = Most likely a serious problem - resolve or explain
0 **ALERT level B** = A potentially serious problem, consider carefully
2 **ALERT level C** = Check. Ensure it is not caused by an omission or oversight
4 **ALERT level G** = General information/check it is not something unexpected

1 ALERT type 1 CIF construction/syntax error, inconsistent or missing data
2 ALERT type 2 Indicator that the structure model may be wrong or deficient
3 ALERT type 3 Indicator that the structure quality may be low
0 ALERT type 4 Improvement, methodology, query or suggestion
0 ALERT type 5 Informative message, check

It is advisable to attempt to resolve as many as possible of the alerts in all categories. Often the minor alerts point to easily fixed oversights, errors and omissions in your CIF or refinement strategy, so attention to these fine details can be worthwhile. In order to resolve some of the more serious problemsit may be necessary to carry out additional measurements or structure refinements. However, the purpose of your study may justify the reported deviations and the more serious of these should normally be commented upon in the discussion or experimental section of a paper or in the "special_details" fields of the CIF. checkCIF was carefully designed to identify outliers and unusual parameters, but every test has its limitations and alerts that are not important in a particular case may appear. Conversely, the absence of alerts does not guarantee there are no aspects of the results needingattention. It is up to the individual to critically assess their own results and, if necessary, seek expert advice.

Publication of your CIF in IUCr journals

A basic structural check has been run on your CIF. These basic checks will be run on all CIFs submitted for publication in IUCr journals (*Acta Crystallographica*, *Journal of Applied Crystallography*, *Journal of Synchrotron Radiation*); however, if you intend to submit to *Acta Crystallographica Section C* or *E* or *IUCrData*, you should make sure that full publication checks arerun on the final version of your CIF prior to submission.

Publication of your CIF in other journals

Please refer to the *Notes for Authors* of the relevant journal for any special instructions relating to CIFsubmission.

PLATON version of 18/12/2021; check.def file version of 18/12/2021

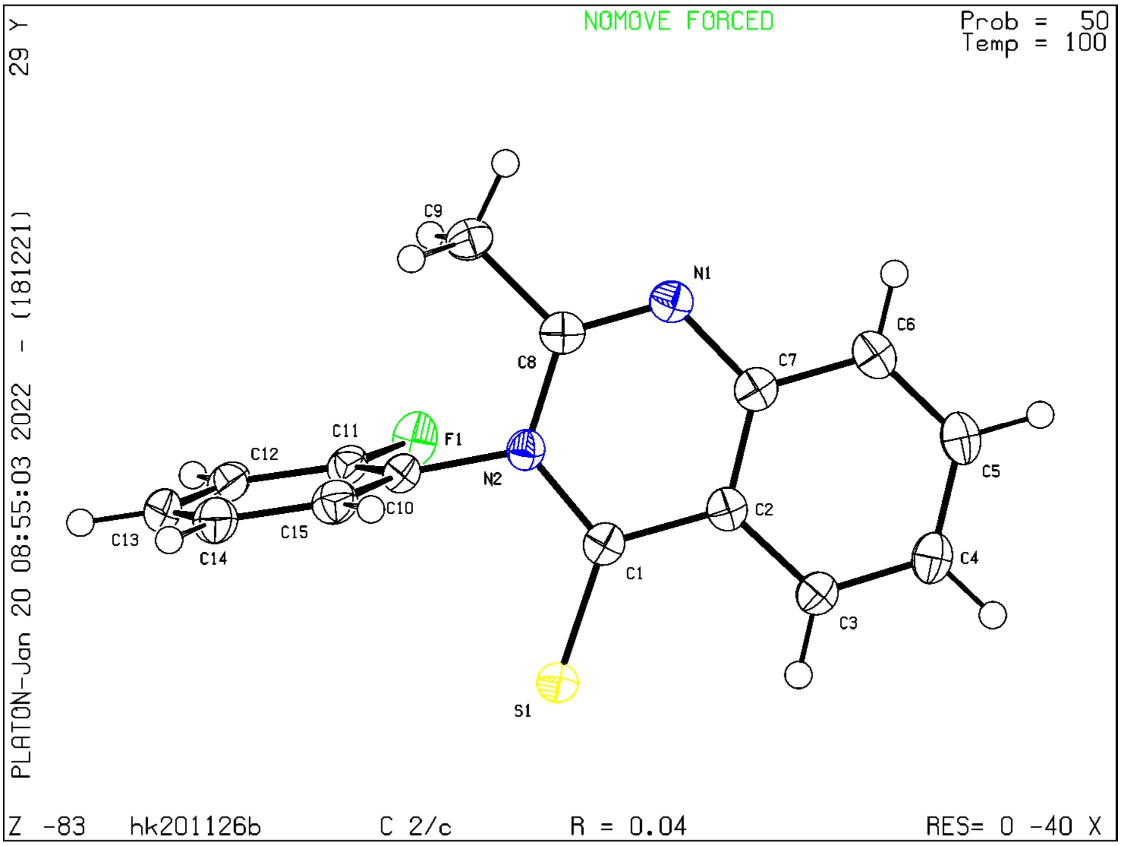

Figure S5. Datablock hk201126b - ellipsoid plot (**rac-2c**).

Hirshfeld Surface analysis

HS analysis showing intermolecular interactions for sulfur and halogen substituents.

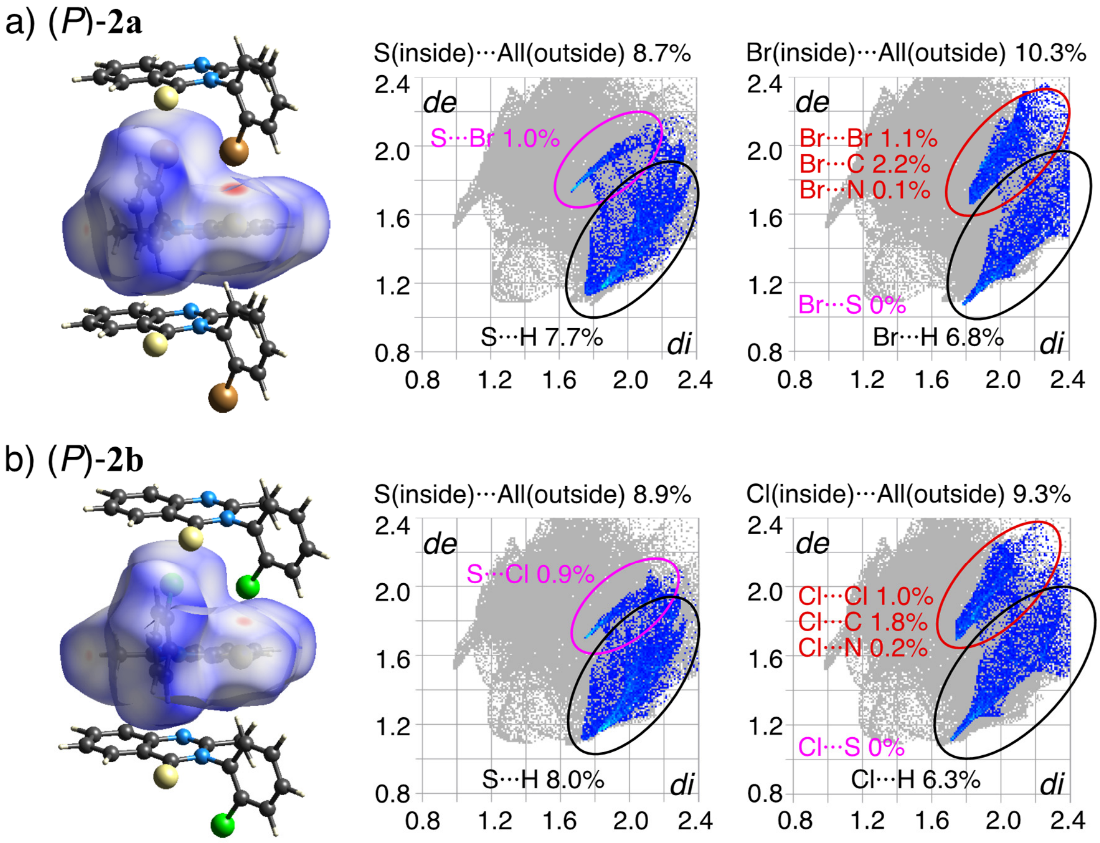

Figure S6. The HS mapped with d_{norm} and the fingerprint plots of enantiomer a) (*P*)-**2a** and b) (*P*)-**2b** showing the d_e and d_i of 0.8-2.4 Å for remarkable interactions [0.5 isovalue of molecular weight function w(r)].

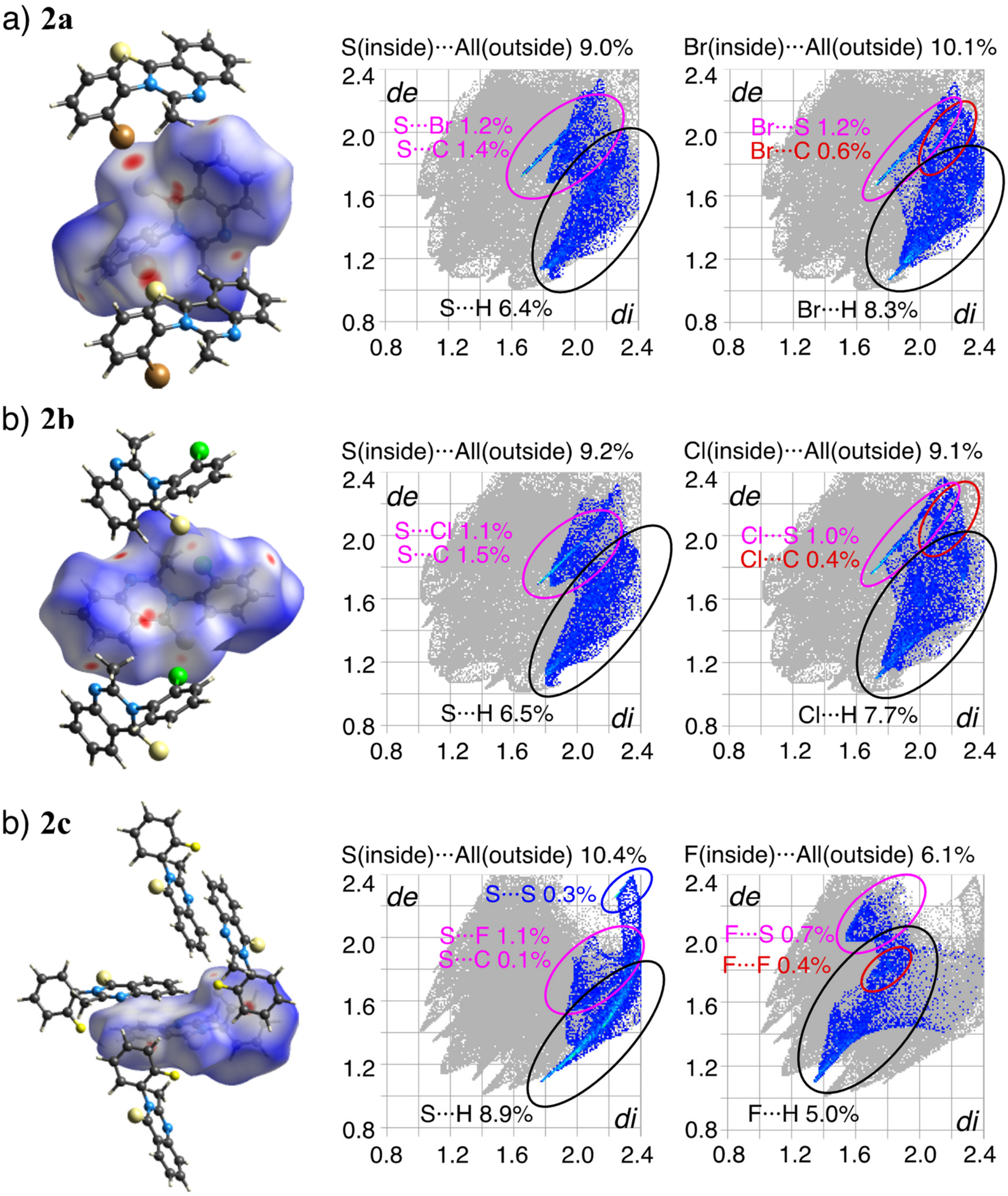

Figure S7. The HS mapped with d_{norm} and the fingerprint plots of racemic a) racemic **2a**, b) racemic **2b**, and c) racemic **2c** showing the d_e and d_i of 0.8-2.4 Å for remarkable interactions [0.5 isovalue of molecular weight function w(r)].
